# Supplementary figures and images for: Rag1 and Rag2 Gene Expressions Identify Lymphopoietic Tissues in Larvae of Rice-Field Eel (Monopterus albus)
Source: Int J Mol Sci. 2022 Jul 7;23(14):7546. doi: 10.3390/ijms23147546 (PMC9324350; doi:10.3390/ijms23147546)

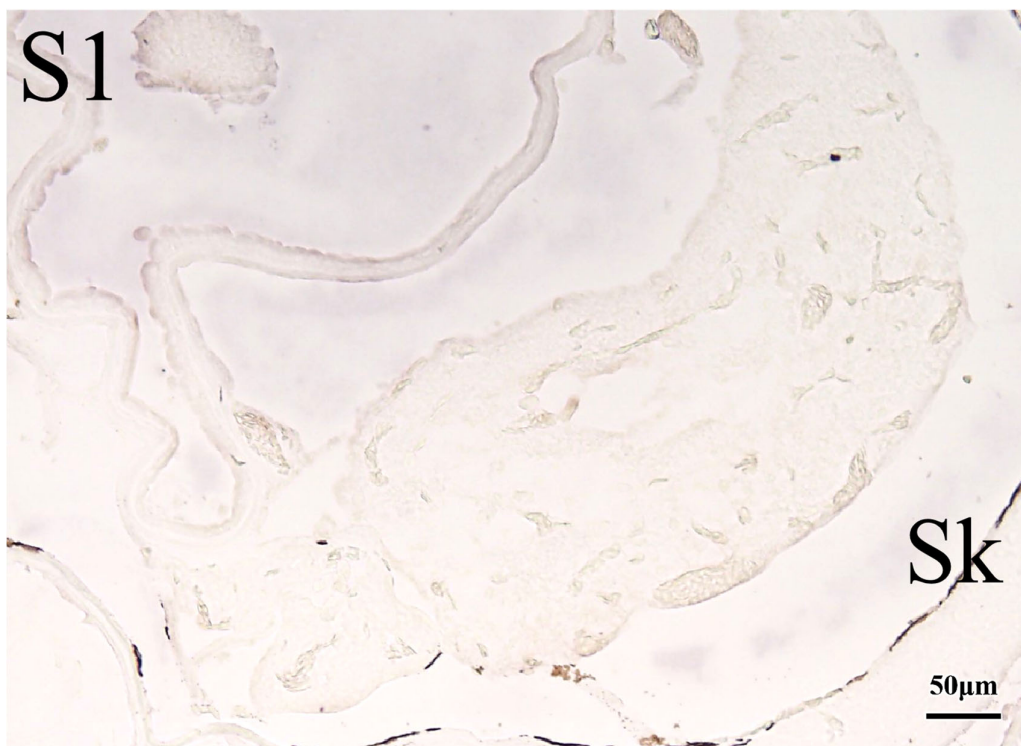

**Figure S1.** Negative control of *rags* gene expression in liver. NBT/BCIP staining. Bars: 50 µm. Sk: skin.

Supplement: Supplementary file 1 [file ijms-23-07546-s001.zip › ijms-1762706-supplementary.pdf]
